# Short- and long-term outcomes in oliguric and non-oliguric acute kidney injury in intensive care: a retrospective, post hoc, bicentric study

**DOI:** 10.1093/ckj/sfaf170

**Published:** 2025-05-30

**Authors:** Sarjit Singh, Mark Andonovic, Jamie P Traynor, Martin F Shaw, Malcolm A B Sim, Patrick B Mark, Kathryn A Puxty

**Affiliations:** Academic Unit of Anaesthesia, Critical Care and Perioperative Medicine, University of Glasgow, Glasgow, UK; Academic Unit of Anaesthesia, Critical Care and Perioperative Medicine, University of Glasgow, Glasgow, UK; Glasgow Renal and Transplant Unit, Queen Elizabeth University Hospital, Glasgow, UK; Academic Unit of Anaesthesia, Critical Care and Perioperative Medicine, University of Glasgow, Glasgow, UK; Academic Unit of Anaesthesia, Critical Care and Perioperative Medicine, University of Glasgow, Glasgow, UK; Department of Intensive Care, Queen Elizabeth University Hospital, Glasgow, UK; Glasgow Renal and Transplant Unit, Queen Elizabeth University Hospital, Glasgow, UK; School of Cardiovascular and Metabolic Health, University of Glasgow, Glasgow, UK; Academic Unit of Anaesthesia, Critical Care and Perioperative Medicine, University of Glasgow, Glasgow, UK; Department of Intensive Care Medicine, Glasgow Royal Infirmary, Glasgow, UK

**Keywords:** acute kidney injury, epidemiology, intensive care, sepsis, survival analysis

## Abstract

**Background:**

Patients admitted to intensive care units (ICUs) frequently develop acute kidney injury (AKI). There is limited research comparing outcomes between oliguric and non-oliguric AKI in this population. This study aimed to investigate the short- and long-term outcomes in oliguric and non-oliguric AKI in intensive care patients; the specific outcomes assessed were mortality and major adverse kidney events. We hypothesised that short- and long-term outcomes would be poorer in oliguric compared with non-oliguric AKI in intensive care patients.

**Methods:**

This retrospective observational cohort study utilised prospectively collected data routinely gathered during patients’ admission. All adult patients >16 years of age admitted to two large Scottish general adult ICUs were included. Patients with long-term kidney replacement therapy, prior transplantation and ICU readmission were excluded. Oliguria was defined as urine output <0.3 ml/kg/h for 24 h. Outcomes were assessed using Cox proportional hazards analyses; should its assumptions be violated, odds ratios at prespecified time points were undertaken.

**Results:**

Of the 2147 patients identified with *de novo* AKI, 1666 had sufficient urine output data for analysis. A total of 528 (31.7%) subjects had oliguric AKI lasting at least 24 h. The 1-year mortality was higher in oliguric patients [adjusted hazard ratio 1.45 (95% confidence interval 1.02–2.12), *E*-value 1.93]. Our data violated the proportional hazards assumption for major adverse kidney events; the 1-year odds ratio for major adverse renal events was non-significant at 1.25 (95% confidence interval 0.92–1.69).

**Conclusion:**

Our study demonstrated that one-third of patients with AKI in intensive care developed oliguria using a standardised definition of oliguria. Oliguric AKI was found to be significantly associated with higher rates of mortality from in-critical care through 1-year post-discharge.

## Graphical Abstract

**Graphical Abstract:**
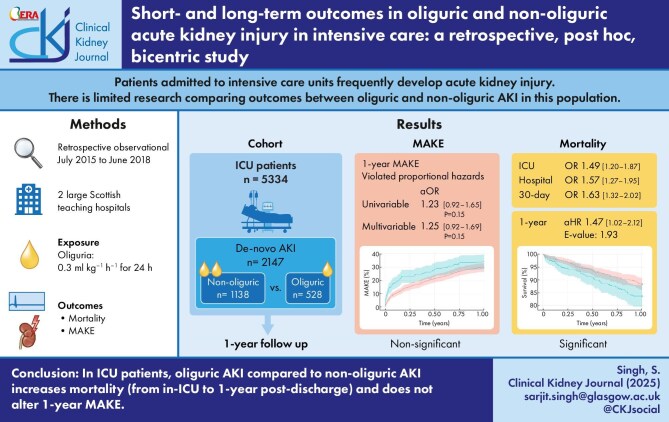

KEY LEARNING POINTS **What was known:**

- Intensive care unit (ICU) patients often develop acute kidney injury (AKI).
- Oliguric AKI is associated with increased morbidity and mortality compared with non-oliguric AKI up to 28-days after hospital discharge (in ICU and non-ICU populations).
- The ICU literature predates the 2012 Kidney Disease: Improving Global Outcomes definition of oliguria, resulting in multiple, heterogenous definitions.

**This study adds:**

- Oliguria (urine output <0.3 ml/kg/h for 24 h) was common in the ICU population, with a prevalence of 31.7%.
- Higher mortality was observed in the oliguric cohort from in-ICU to 1 year after hospital discharge [adjusted hazard ratio 1.45 (95% CI 1.02–2.12), *E*-value 1.93].
- Oliguric AKI did not alter the risk of 1-year major adverse renal events.

**Potential impact:**

- Further work is required to assess if this increased mortality is observed beyond 1 year.
- Patients developing oliguric AKI may warrant additional or earlier intervention in critical care or in hospital.
- This cohort of patients may benefit from longer-term follow-up or surveillance after ICU discharge.

## INTRODUCTION

Acute kidney injury (AKI) is defined by an increase in serum creatinine and/or oliguria/anuria [1]. Patients admitted to intensive care units (ICUs) frequently develop AKI, with a prevalence of 30–57% [2–4]. AKI in patients admitted to the ICU has been shown to worsen short-term and long-term outcomes with increases noted in both in-hospital and 1-year mortality [5]. Limited research has shown patients admitted to the ICU have increased the risk of both long-term mortality and progression to chronic kidney disease (CKD) [5, 6].

There is evidence to suggest that oliguric and non-oliguric AKI have differing impacts in the short and long term [7, 8]. Oliguric AKI has been shown to be associated with higher mortality and morbidity in the non-ICU hospital population [7, 8].

Previously, Mandelbaum *et al.* [9] assessed 17 227 ICU patients with AKI for in-hospital outcomes based on the presence or absence of oliguria. They found that oliguric AKI had a higher in-hospital mortality compared with non-oliguric AKI.

Two prior studies have looked at oliguric and non-oliguric ICU patients requiring kidney replacement therapy (KRT) for AKI [10, 11]. Wald *et al.* [10] assessed 85 ICU patients requiring KRT and looked at in-hospital mortality. They found that oliguric patients (*n* = 51) had poorer outcomes compared with non-oliguric patients (in-hospital mortality 69.8% versus 45.2%, *P* = .026). A 2013 study looked at 361 ICU patients receiving KRT and assessed 28-day mortality [11]. This study observed lower mortality in the non-oliguric group [odds ratio (OR) 0.85 (95% CI 0.65–0.99)].

Kidney Disease: Improving Global Outcomes (KDIGO) defined oliguric AKI in 2012 and most studies are from before this international definition was widely adopted and therefore variation exists within the literature. Mandelbaum *et al.* [9] published their study in 2013 but looked at oliguria as a continuum rather than using a binary definition. Previous work from 2006 defined oliguria as <400 ml over 24 h [6], whereas a subsequent study by Oh *et al.* [11] defined oliguria as <107 ml over 6 h (equating to 428 ml/24 h).

This study aimed to investigate the short- and long-term outcomes in oliguric and non-oliguric AKI in ICU patients. The specific outcomes for the study were mortality and major adverse kidney events (MAKEs).

## MATERIALS AND METHODS

This cohort study utilised data routinely gathered during patients’ ICU admission and follow-up period, therefore individualised participant consent was not sought; ethical approval was granted by the National Health Services London-Surrey Research and Ethics Committee (Ref: 18/LO/2060) prior to commencement. Findings are reported based on Strengthening the Reporting of Observational Studies in Epidemiology (STROBE) guidelines [12].

Details of the study methods are described elsewhere [4]. In brief, all adult patients admitted to two large Scottish general adult ICUs between 1 July 2015 and 30 June 2018 were included. Patients with long-term kidney replacement therapy (KRT), prior kidney transplantation and readmission to the ICU over the total study period were excluded. Patients without AKI, urine output or outcome data were excluded.

KDIGO guidelines were used to define oliguria (urine output <0.3 ml/kg/h for 24 h) [1]. All patients had urine output data collected and recorded hourly while in the ICU, regardless of whether a urinary catheter was *in situ*; to provide complete representation of the general ICU population, we did not look solely at catheterised patients. We synthesised urine output into 24-h values and compared these to KDIGO-based patient-specific thresholds (0.3 ml/kg/h for 24 h). We did not use the second KDIGO oliguria definition (<0.5 ml/kg/h for 6 h) to define patients as oliguric, as this would incorrectly label non-catheterised patients as oliguric if they slept for >6 h. However, as a sensitivity analysis, we applied this definition of oliguria to patients to assess for potential misclassification bias. Oliguria was not assessed on the day of ICU admission or discharge, as these days would have incomplete 24-h measurements. Oliguria was defined first using actual body weight (ABW) and again using predicted body weight (PBW), as no consensus exists on which should be used in the context of the ICU [13–15]. Given the differences between ABW and PBW are poorly studied, we compared both ABW and PBW, but primarily used ABW, as this yields increased sensitivity for detection of oliguria [15].

‘ICU survivors’ were defined as patients alive 30 days after hospital discharge to mitigate the impact of patients discharged from hospital for end-of-life care. MAKEs could occur any day after becoming an ICU survivor and was defined as a decrease of at least 30% in estimated glomerular filtration rate (eGFR) from baseline, doubling of serum creatinine and initiation of long-term KRT [16, 17]. MAKEs were captured automatically by the Vitalpulse Strathclyde Electronic Renal Patient Record (SERPR) database. The SERPR covers >2.6 million patients and is used to document all interactions with nephrology services (including data on KRT) and automatically retrieves laboratory, radiology and demographic data from primary, secondary and tertiary care across six Scottish health boards.

Pre-admission serum creatinine results were used to calculate a baseline value for each patient: a validated automated system was used to select the most appropriate reference, comprising either the median value from 8 to 365 days before admission or the lowest value in the week prior to admission [18]; if both values were available then the median value from 8 to 365 days was preferentially selected. Additionally, this reference value was used to calculate baseline eGFR using the Chronic Kidney Disease Epidemiology Collaboration equation [16]. The reference value was then used to diagnose *de novo* kidney injury using the KDIGO classification. The initial injury was identified as the point at which AKI criteria were met for the first time. Days during which patients received KRT in the ICU were identified from the Scottish Intensive Care Society Audit Group (SICSAG) database, and this superseded serum creatinine values on the days this support was delivered; serum creatinine values within 24 h of discontinuation of KRT were also discounted to avoid artificially lowered creatinine from suggesting early kidney recovery. Severity of injury was classified using the worst creatinine value from the duration of the injury or delivery of KRT.

Pre-existing comorbidities were identified using data in Philips Carevue electronic patient records and grouped according to cardiovascular disease (including chronic hypertension and ischaemic heart disease), respiratory disease (including obstructive airway disease and interstitial lung disease), liver disease (including alcoholic and non-alcoholic fatty liver disease, hepatitis or cirrhosis) and diabetes mellitus (of all types regardless of severity of disease). Admission diagnoses were determined by the physicians admitting and discharging the patient from the ICU and organised into 26 broad groups based on coding in the SICSAG database [4].

All statistical analysis was performed using R version 4.0.5 (R Foundation for Statistical Computing, Vienna, Austria). Age and eGFR were handled as categorical variables, as their effect was likely non-linear; aging 1 year as an 18-year-old is biologically different from aging 1 year as an 80-year-old. Variables were summarised using median values and interquartile range (IQR) or by proportions with 95% confidence interval (CI). Differences in median values were compared using the Wilcoxon rank-sum test, whereas differences in proportions were compared using the Pearson chi-squared test. Mortality was assessed to ICU discharge, hospital discharge and day 30 after hospital discharge. We attempted to analyse our data using Cox proportional hazards (CPH) modelling should no assumptions be violated. Should data not meet the assumptions, chi-squared tests and ORs were assessed. As a sensitivity analysis, stratified CPH were undertaken to aid triangulation of our results. Analyses were stratified into patients with oliguric and non-oliguric AKI. Initial univariable analyses were performed on each collected variable with the exception of Acute Physiology and Chronic Health Evaluation II (APACHE II) scores; this avoided collinearity, as urine output is used in their calculation. Univariable *P*-values of <0.2 were included in the multivariable model to avoid removing a variable that may subsequently become significant on multivariable modelling. For all adjusted analyses, statistical significance was set at a two-sided *P*-value <.05. Directed acyclic graphs (DAGs) are supplied in the supplementary material to further explain which confounders were adjusted for. Based on these, we undertook mediation analysis for APACHE II score and ICU length of stay, as these may mediate the effect of oliguria on outcomes.

## RESULTS

**Figure 1:**
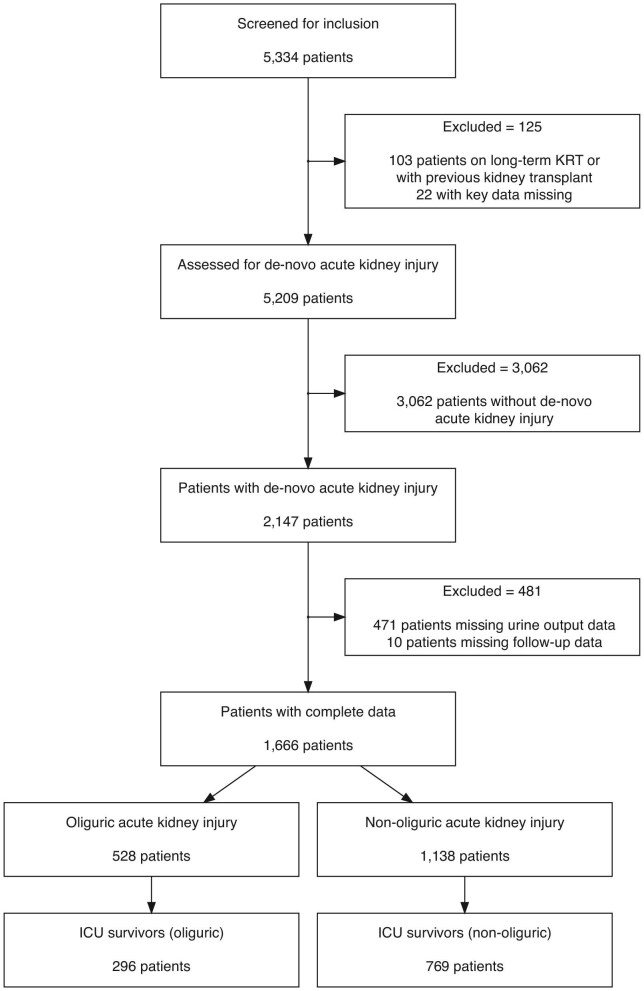
STROBE flow diagram.

A total of 5334 patients were found in our search of the SICSAG database (Fig. 1). Of these, 1666 (31.2%) patients had *de novo* AKI with available urine output values. The median 24-h urine output from across the study population was 1185 ml (IQR 375–1772). These values were then corrected individually for patient weights [median 15.7 ml/kg/24 h (IQR 5.2–25.2 ml/kg/24 h)]. The median urine output in the oliguric group was found to be significantly lower than in the non-oliguric group (4.7 versus 19.4 ml/kg/24 h, *P* < .001). Baseline characteristics stratified by the presence of oliguria are presented in Table 1.

**Table 1:** Cohort demographics.

Characteristics	Non-oliguric (*n* = 1138)	Oliguric (*n* = 528)	*P*-value
Male, *n* (%)	669 (58.8)	333 (63.1)	.108
Age (years), median (IQR)	61.00 (48.00–72.00)	61.50 (50.00–71.00)	.337
Baseline eGFR (ml/min/1.73 m^2^), median (IQR)	85.11 (63.73–100.74)	78.90 (51.84–96.91)	<.001
Pre-existing comorbidities, *n* (%)			
Cardiovascular disease	492 (43.2)	250 (47.3)	.129
Respiratory disease	257 (22.6)	99 (18.8)	.087
Liver disease	124 (10.9)	63 (11.9)	.589
Diabetes mellitus	189 (16.6)	115 (21.8)	.013
Cancer	85 (7.5)	34 (6.5)	.594
Admission from surgical specialty, *n* (%)	579 (50.9)	288 (54.5)	.18
Admission with sepsis, *n* (%)	341 (30.0)	190 (36.0)	.017
APACHE II score, median (IQR)	21.00 (15.00–27.00])	25.00 (20.00–30.00)	<.001
ACP level 3 (days), median (IQR)	2.00 (1.00–6.00)	5.00 (3.00–12.00)	<.001
ACP level 2 (days), median (IQR)	1.00 (0.00–2.00)	1.00 (0.00, 3.00)	.01
Renal support			
Proportion supported, *n* (%)	102 (9.0)	289 (54.7)	<.001
Days, median (IQR)	2.00 (1.00–2.00)	5.00 (3.00–9.00)	<.001
Ventilatory support			
Proportion supported, *n* (%)	861 (75.7)	409 (77.5)	.476
Days, median (IQR)	3.00 (2.00–7.00)	6.00 (3.00–14.00)	<.001
Vasopressor support			
Proportion supported, *n* (%)	771 (67.8)	421 (79.7)	<.001
Days, median (IQR)	2.00 (1.50–4.50)	4.00 (2.00–8.00)	<.001
Length of renal injury (days), median (IQR)	1.00 (1.00–3.00)	4.00 (2.00–14.00)	<.001
Length of ICU stay (days), median (IQR)	3.00 (1.00–7.00)	6.00 (3.00–14.00)	<.001
Length of hospital stay (days), median (IQR)	12.00 (5.00–25.00)	16.00 (7.00–31.00)	<.001

ACP: augmented care period.

### Patient characteristics

A total of 528 (31.7%) subjects had oliguric AKI lasting at least 24 h. Patients who developed oliguric AKI had a lower baseline eGFR [78.9 ml/min/1.73 m^2^ (IQR 51.8–96.9) versus 85.1 ml/min/1.73 m^2^ (IQR 63.7–100.7), *P* < .001]. A higher proportion of patients with oliguric AKI were admitted with sepsis (36.0% versus 30.0%. *P* = .017) and had higher admission APACHE II scores (24.91 versus 21.28, *P* < .001).

Oliguric patients had AKI that lasted longer compared with non-oliguric AKI [median 4 h (IQR 2–14) versus 1 h (IQR 1–3), *P* < .001]. Oliguric patients more commonly received KRT (54.7% versus 9.0%, *P* < .001) and had a longer median duration of KRT [median 2 days (IQR 0–6) versus 5 days (IQR 3–12), *P* < .001]. Length of stay was longer in oliguric patients both in the ICU [median 3 days (IQR 1–7) versus 6 days (IQR 3–14), *P* < .001] and in hospital [median 12 days (IQR 5–25) versus 16 days (IQR 7–31), *P* < .001].

Supplementary Table U1 presents the characteristics of patients missing urine output data. These patients were broadly similar but had shorter ICU stays and fewer days on ventilatory and vasopressor support and, in turn, lower levels of augmented care period support. These patients also had lower rates of sepsis and lower APACHE II scores. Of the 1666 patients included in the study population, 92 had at least one documented hourly output value registered as ‘self-void’ as opposed to via a catheter. However, on further analysis of these patients, none of these hourly values occurs on the day of their lowest urine output (and the value that was used to define them as oliguric or non-oliguric).

As a sensitivity analysis, we attempted to classify patients as oliguric using a rolling 6-h window using a cut-off of 0.5 ml/kg/h. This reclassified an additional 92 patients as oliguric; however, on detailed interrogation of the data, the majority [*n* = 75 (81.5%)] of this reclassification occurred overnight between the hours of 21:00 and 07:00, in keeping with normal physiology rather than a pathological process.

### Mortality

**Figure 2:**
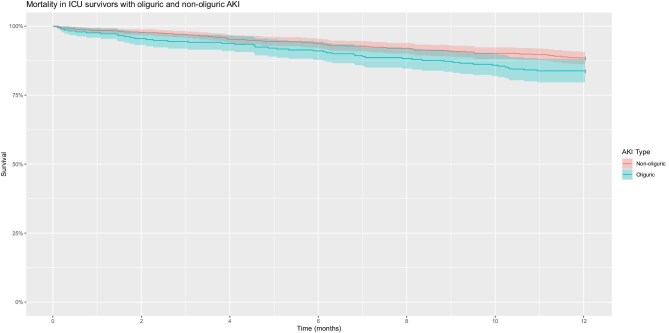
Mortality in ICU survivors with oliguric and non-oliguric AKI.

Mortality outcomes are shown in Table 2 and Fig. 2. Mortality was higher in oliguric AKI at all assessed time points up to 30 days after hospital discharge. The OR for in-ICU mortality in oliguric AKI was 1.49 (95% CI 1.20–1.87), which increased to 1.57 (95% CI 1.27–1.95) for in-hospital mortality and further increased to 1.63 (95% CI 1.32–2.02) for mortality within 30 days of hospital discharge. For all aforementioned time points, chi-squared tests all reached statistical significance (*P* < .001).

**Table 2:** ORs for mortality.

Mortality	Non-oliguric (*n* = 1138)	Oliguric (*n* = 528)	*P*-value	OR (95% CI)
Death in ICU	296	182	<.001	1.49 (1.20–1.87)
Death in hospital	357	221	<.001	1.57 (1.27–1.95)
Death within 30 days of hospital discharge	369	232	<.001	1.63 (1.31–2.02)

Figure 2 is a Kaplan–Meier plot for mortality in ICU survivors stratified by oliguric and non-oliguric AKI; divergence occurred between oliguric and non-oliguric strata. The 1-year mortality met the assumptions of the CPH. The 95% CIs of our Kaplan–Meier plot overlapped at 1 year: oliguric 95% CI 79.7–88.4% and non-oliguric 95% CI 86.2–90.8%.

Table 3 presents both unadjusted and adjusted CPH models. The model was adjusted in three additive steps to minimise the risk of introducing bias. Across all four models, oliguria was associated with a statistically significant increase in mortality, with a fully adjusted hazard ratio (HR) of 1.47 (95% CI 1.02–2.12, *P* = .038). The *E*-value for this result was 1.93 (common outcome assumption) and increased to 2.30 (rare outcome assumption). Table 3 also includes mediation analysis, indicating that the effect was partially mediated by length of stay (20.7%) and APACHE II score (18.4%).

**Table 3:** CPH modelling and mediation analysis in ICU survivors.

Cox proportional hazard modelling for mortality in ICU survivors												
	Unadjusted			Partially adjusted 1			Partially adjusted 2			Fully adjusted		
Characteristics	HR	95% CI	*P*-value	HR	95% CI	*P*-value	HR	95% CI	*P*-value	HR	95% CI	*P*-value
AKI type												
Non-oliguric	Ref	–		Ref	–		Ref	–		Ref	–	
Oliguric	1.45	1.02–2.06	.041	1.44	1.00–2.06	.048	1.45	1.01–2.08	.043	1.47	1.02–2.12	.038
Age (years)												
>30				Ref	–		Ref	–		Ref	–	
30–60				1.68	0.90–3.13	.101	1.59	0.85–2.97	.142	1.63	0.87–3.04	.127
>60				2.07	1.11–3.87	.023	1.86	0.99–3.50	.055	1.91	1.01–3.61	.047
Baseline eGFR (ml/min/1.73 m^2^)												
>60				Ref	—		Ref	—		Ref	—	
30–60				1.07	0.68–1.68	.775	1.04	0.66–1.65	.865	1.05	0.66–1.66	.843
<30				0.87	0.43–1.73	.685	0.83	0.42–1.68	.612	0.84	0.42–1.69	.631
Admission with sepsis				0.97	0.67–1.39	.854	0.97	0.67–1.39	.863	0.99	0.68–1.43	.940
Pre-existing diabetes							1.57	1.06–2.33	.024	1.57	1.06–2.32	.025
Pre-existing cancer							2.00	1.19–3.36	.009	2.05	1.21–3.47	.008
Vasopressor support										0.89	0.60–1.32	.568
Ventilatory support										1.13	0.76–1.66	.550
Mediation analysis for length of ICU admission												
Controlled direct effect	1.13	0.59–1.72		1.06	0.59–1.61		1.08	0.59–1.65		1.09	0.60–1.62	
Natural direct effect	1.34	0.88–1.88		1.34	0.82–1.83		1.36	0.83–1.96		1.54	0.73–2.01	
Natural indirect effect	1.07	0.99–1.28		1.09	1.00–1.30		1.10	1.01–1.32		1.09	1.01–1.31	
Total effect	1.43	0.95–2.02		1.46	0.92–2.03		1.50	0.97–2.15		1.68	0.84–2.29	
Proportion mediated, %	20.8	–		26.7	–		26.6	–		20.7	–	
Mediation analysis for APACHE II score												
Controlled direct effect	1.56	0.58–4.16		1.39	0.45–3.97		1.56	0.50–4.24		1.61	0.49–4.38	
Natural direct effect	1.30	0.91–1.85		1.34	0.92–1.90		1.41	0.86–1.96		1.42	0.89–2.15	
Natural indirect effect	1.10	0.99–1.24		1.08	0.98–1.20		1.07	0.97–1.19		1.07	0.98–1.18	
Total effect	1.44	1.01–1.98		1.45	0.99–2.05		1.50	0.94–2.10		1.51	0.95–2.26	
Proportion mediated, %	30.3	–		24.1	–		18.9	–		18.4	–	

Table 4 shows univariable and multivariable logistic regression models for mortality in ICU survivors at 1 year. Oliguria was not statistically significant in our univariable [OR 1.46 (95% CI 0.99–2.13), *P* = .055] and multivariable [OR 1.44 (95% CI 0.97–2.11), *P* = .066] logistic regression for mortality.

**Table 4:** Logistic regression for 1-year mortality.

	Univariable		Multivariable	
Characteristics	OR (95% CI)	*P*-value	OR (95% CI)	*P*-value
AKI type				
Non-oliguric	Ref		Ref	
Oliguric	1.46 (0.99–2.13)	.055	1.44 (0.97–2.11)	.066
Age (years)				
<30	Ref		Ref	
30–60	1.77 (0.96–3.52)	.084	1.62 (0.87–3.24)	.148
>60	2.19 (1.19–4.39)	.018	1.90 (1.02–3.82)	.056
Sex				
Female	Ref			
Male	1.16 (0.80–1.70)	.432		
Baseline eGFR (ml/min/1.73 m^2^)				
>60	Ref			
30–60	1.22 (0.74–1.94)	.419		
<30	0.98 (0.44–1.93)	.951		
Admitting specialty				
Medical	Ref			
Surgical	0.85 (0.59–1.22)	.369		
Admission diagnosis				
Non-sepsis	Ref			
Sepsis	1.01 (0.68–1.47)	.977		
Cardiovascular comorbidities				
None	Ref			
Pre-existing diagnosis	1.08 (0.75–1.56)	.672		
Pre-existing diabetes				
None	Ref		Ref	
Pre-existing diagnosis	1.71 (1.11–2.58)	.013	1.68 (1.09–2.56)	.017
Respiratory comorbidities				
None	Ref			
Pre-existing diagnosis	1.16 (0.74–1.77)	.502		
Pre-existing liver disease				
None	Ref			
Pre-existing diagnosis	1.28 (0.69–2.24)	.401		
Pre-existing cancer				
None	Ref		Ref	
Pre-existing diagnosis	2.11 (1.16–3.67)	.011	2.13 (1.15–3.75)	.012

Ref: reference.

### MAKEs

**Figure 3:**
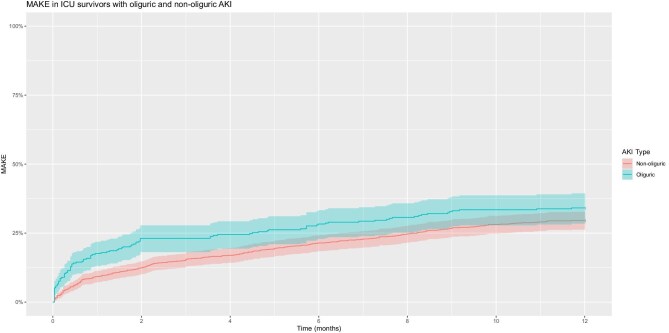
MAKEs in ICU survivors with oliguric and non-oliguric AKI.

Figure 3 shows Kaplan–Meier plots for MAKEs in ICU survivors with oliguric and non-oliguric AKI. MAKEs violated the proportional hazards assumption so was deemed unsuitable for analysis with CPH or Fine and Gray subdistribution hazards modelling (Supplementary Fig. 1; Schoenfeld residuals *P* = .0002). Instead, MAKE was analysed using ORs at 1 year [OR 1.23 (95% CI 0.93–1.65), *P* = .151]. Table 5 shows univariable and multivariable logistic regression models for MAKEs at 1 year. Oliguria did not reach statistical significance in either univariable [OR 1.23 (95% CI 0.92–1.65), *P* = .152] or multivariable [OR 1.25 (95% CI 0.92–1.69), *P* = .153] analysis.

**Table 5:** Logistic regression for 1-year MAKEs.

	Univariable		Multivariable	
Characteristics	OR (95% CI)	*P*-value	OR (95% CI)	*P*-value
AKI type				
Non-oliguric	Ref		Ref	
Oliguric	1.23 (0.92–1.65)	.152	1.25 (0.92–1.69)	.153
Age (years)				
<30	Ref		Ref	
30–60	3.43 (2.10–5.91)	<.001	2.79 (1.68–4.87)	<.001
>60	4.36 (2.65–7.53)	<.001	3.29 (1.92–5.90)	<.001
Sex				
Female	Ref		Ref	
Male	0.57 (0.44–0.75)	<.001	0.63 (0.47–0.83)	.001
Baseline eGFR (ml/min/1.73 m^2^)				
>60	Ref		Ref	
30–60	1.60 (1.13–2.26)	.008	1.26 (0.87–1.81)	.219
<30	0.60 (0.32–1.05)	.085	0.46 (0.24–0.83)	.014
Admitting specialty				
Medical	Ref			
Surgical	0.93 (0.72–1.22)	.610		
Admission diagnosis				
Non-sepsis	Ref		Ref	
Sepsis	1.40 (1.06–1.84)	.016	1.31 (0.98–1.74)	.067
Cardiovascular comorbidities				
None	Ref		Ref	
Pre-existing diagnosis	1.51 (1.16–1.97)	.002	1.16 (0.87–1.56)	.316
Pre-existing diabetes				
None	Ref		Ref	
Pre-existing diagnosis	2.12 (1.53–2.92)	<.001	1.98 (1.41–2.77)	<.001
Respiratory comorbidities				
None	Ref		Ref	
Pre-existing diagnosis	1.30 (0.94–1.77)	.107	1.08 (0.77–1.50)	.646
Pre-existing liver disease				
None	Ref		Ref	
Pre-existing diagnosis	1.36 (0.88–2.10)	.161	1.44 (0.91–2.27)	.116
Pre-existing cancer				
None	Ref			
Pre-existing diagnosis	1.26 (0.76–2.04)	.358		

Ref: reference.

As a sensitivity analysis, we undertook stratified CPH modelling. Using a cut-off of 0.5 months in a stratified model, the proportional hazards assumption was met (Supplementary Fig. 1; Schoenfeld residuals *P* = .2719). With this model, the HR gained statistical significance [HR 2.41 (95% CI 1.58–3.66), *P* < .001; Supplementary Table C1). The time strata interaction term for period 2, i.e. beyond the first 0.5 months of the model, was 0.39 (95% CI 0.23–0.65, *P* < .001). To assess the HR in period 2, we analysed period 2 in isolation (Supplementary Table C2), which yielded a non-significant HR [0.94 (95% CI 0.70–1.26), *P* = .7). This is concordant with the results from the stratified CPH model estimate of 0.94 (i.e. 2.41 × 0.39). The overall HRs for this model are HR 2.41 (95% CI 1.58–3.66, *P* < .001) from 0 to 0.5 months and HR 0.94 (95% CI 0.70–1.26, *P* = .7) from 0.5 to 12 months. This is concordant with our assessment using ORs showing no difference in the 1-year MAKE risk between oliguric and non-oliguric AKI.

### Risk factors for oliguria

Table 6 shows univariable and multivariable logistic regression models for oliguria. Baseline eGFR <30 ml/min/1.73 m^2^ increased the risk of oliguria in both univariable and multivariable analysis [multivariable OR 2.27 (95% CI 1.53–3.37), *P* < .001]. Admissions due to sepsis were associated with an increased risk of oliguria [multivariable OR 1.33 (95% CI 1.06–1.66), *P* = .013], as did admission from a surgical specialty [multivariable OR 1.25 (95% CI 1.01–1.55), *P* = .039]. Pre-existing comorbidities also did not reach statistical significance for affecting oliguria risk. The exception to this was diabetes in the univariable analysis [OR 1.40 (95% CI 1.08–1.81), *P* = .011], but this was lost in the multivariable analysis.

**Table 6:** Logistic regression for development of oliguria.

	Univariable		Multivariable	
Characteristics	OR (95% CI)	*P*-value	OR (95% CI)	*P*-value
Age (years)				
<30	Ref		Ref	
30–60	1.48 (1.05–2.11)	.029	1.47 (1.03–2.12)	.035
>60	1.48 (1.05–2.11)	.029	1.38 (0.95–2.04)	.096
Sex				
Female	Ref		Ref	
Male	1.19 (0.97–1.48)	.101	1.22 (0.98–1.53)	.070
Baseline eGFR (ml/min/1.73 m^2^)				
>60	Ref		Ref	
30–60	1.25 (0.96–1.63)	.098	1.19 (0.90–1.57)	.220
<30	2.36 (1.60–3.47)	<.001	2.27 (1.53–3.37)	<.001
Admitting specialty				
Medical	Ref		Ref	
Surgical	1.16 (0.94–1.43)	.159	1.25 (1.01–1.55)	.039
Admission diagnosis				
Non-sepsis	Ref		Ref	
Sepsis	1.31 (1.05–1.63)	.015	1.33 (1.06–1.66)	.013
Cardiovascular comorbidities				
None	Ref		Ref	
Pre-existing diagnosis	1.18 (0.96–1.46)	.112	1.05 (0.84–1.32)	.660
Pre-existing diabetes				
None	Ref		Ref	
Pre-existing diagnosis	1.40 (1.08–1.81)	.011	1.29 (0.98–1.68)	.067
Respiratory comorbidities				
None	Ref		Ref	
Pre-existing diagnosis	0.79 (0.61–1.03)	.081	0.81 (0.62–1.06)	.126
Pre-existing liver disease				
None	Ref			
Pre-existing diagnosis	1.11 (0.80–1.52)	.537		
Pre-existing cancer				
None	Ref			
Pre-existing diagnosis	0.85 (0.56–1.27)	.445		

Ref: reference.

### ABW and PBW

A total of 1079 subjects (64.8% of our total sample size) had data for both ABW and PBW. When determined by PBW rather than ABW, 11 patients were reclassified as oliguric. Conversely, when determined by ABW rather than PBW, 65 patients were reclassified as oliguric.

When assessed using PBW rather than ABW, mortality lost significance on logistic regression [univariable OR 1.33 (95% CI 0.80–2.17), *P* = .260; multivariable OR 1.27 (95% CI 0.76–2.09), *P* = .349] and CPH modelling [HR 1.33 (95% CI 0.83–2.10), logrank *P* = .20]. MAKE remained unchanged when PBW was used.

### KRT

We compared oliguric and non-oliguric AKI in those who did not receive any acute KRT. Although there were fewer patients available for analysis (*n* = 1274), mortality remained greater from in-ICU to in-hospital and 30 days after hospital discharge in the oliguric cohort (Supplementary Table R3). A total of 895 patients were available to analyse 1-year outcomes. Due to this reduction in sample size, 1-year outcomes were not statistically significant but still trended to be poorer in the oliguric cohort [1-year mortality HR 1.15 (95% CI 0.87–1.89) and 1-year MAKE OR 1.16 (95% CI 0.78–1.72); Supplementary Figs. R1 and R2].

## DISCUSSION

We hypothesised that oliguric AKI would confer poorer short- and long-term outcomes compared with non-oliguric AKI in ICU patients. We observed that oliguric AKI is common in ICU patients. In-ICU, in-hospital and 1-year mortality were significantly higher in oliguric patients, however, oliguria did not increase the risk of 1-year MAKEs.

Our study demonstrated that one-third of patients with AKI in the ICU developed oliguria. Definitions of oliguria vary in the literature, ranging from a set value [10] to a continuum [9], regardless of patient characteristics such as weight. In this study, we defined oliguria as urine output <7.2 ml/kg/day (derived from the KDIGO definition of urine output <0.3 ml/kg/h). This creates an individualised threshold for oliguria rather than what has previously been used in the literature.

There is no consensus on the use of consecutive or mean hourly urine output in defining oliguria [14]; the importance of the context surrounding oliguria cannot be understated [13]. We used 24-h urine output, as this averages out urine output and decreases the inaccuracies associated with using routinely collected data (e.g. decreased urine output due to a blocked catheter). It is well documented in the literature that only mean urine output can be used in patients without a urinary catheter [14]; use of consecutive hourly urine output would misclassify significant numbers of non-catheterised patients as oliguric every night while they sleep. Alternately, if we looked solely at patients with urinary catheters *in situ*, we would exclude significant numbers of patients, creating a study grossly misrepresentative of the general ICU population.

This study observed an oliguria rate lower than in previous work, with 31.7% of patients developing oliguric AKI compared with 30–57% reported in the literature [2–4]. These studies looked solely at patients who received KRT and used older definitions of oliguria. This is in keeping with the results from this study, which found significantly higher rates of KRT in the oliguric group.

Comparing oliguric to non-oliguric patients, the median age between groups was similar. However, older patient groups were more likely to develop oliguria. This may be related to older patients having kidneys that are more vulnerable to acute insult due to progressive glomerulosclerosis and loss of functional kidney units with advancing age [19]. Conversely, more patients in the oliguric cohort had pre-existing diabetes mellitus, but this was not significant on multivariable analysis for development of oliguria.

Lower median pre-admission eGFRs were seen in the oliguric cohort, likely reflecting underlying tubular damage and kidneys more vulnerable to an acute insult. On logistic regression, lower baseline eGFRs, particularly <30 ml/min/1.73 m^2^, were significantly associated with oliguric AKI. This is unsurprising given that this corresponds with CKD stages 4 and 5, reflecting kidneys with pre-existing disease and less physiological reserve against acute insult [20].

Admission from surgical specialties and with sepsis were associated with the development of oliguria. The mechanism of these associations is unclear, but it has been previously suggested that the systemic inflammatory response due to surgery or infection may cause damage at the cellular level [21]. Endotoxaemia has been shown in animal models to cause swelling in the proximal convoluted tubule, leading to reduced tubular flow [21]. Another postulated mechanism may be systemic inflammatory mediators causing mitochondrial dysfunction [22]. This is in addition to inflammatory responses leading to systemic vasoplegia and kidney hypoperfusion.

Higher rates of multi-organ support, admissions from surgical specialties and with sepsis were all associated with an increased risk of developing oliguric AKI. Higher rates of cardiovascular support and KRT were received in oliguric patients. Receipt of multi-organ support was also more common, suggestive of more deranged physiology and severe pathology mandating critical care admission [23].

Oliguric AKI was found to be significantly associated with higher rates of in-ICU and in-hospital mortality. Previous work looking at ICU patients receiving KRT and found that in-patient mortality was higher in oliguric AKI [10]. Oh *et al.* found a 15% relative risk reduction in 28-day all-cause mortality in critically ill patients with non-oliguric AKI compared with oliguric AKI. This study echoes the literature that higher mortality is seen in-ICU and in-hospital in oliguric compared with non-oliguric cohorts.

We observed increased mortality in oliguric patients from in-ICU to 1-year after hospital discharge. This increased mortality was observed in CPH analysis but not logistic regression modelling. This is likely due to the CPH having greater sensitivity (CPH factors in the time each patient was ‘at-risk’ prior to an event occurring, unlike logistic regression, which assesses purely if the event occurred or not) [24]. Moreover, survival is rarely normally distributed, making it better suited for analyses with CPH, should the assumptions be met [24].

Of interest, the CIs on our mortality Kaplan–Meier plot cross at 1 year, yet the logrank test at 1 year attained significance (*P* = .04). This apparent contradiction can be explained by overlapping CIs not always reflecting *P* > .05 (point 21, as explained by Altman *et al.*) [25]. When undertaking Kaplan–Meier analyses, the 95% CIs widen over time as removing events/censoring removes patients from the ‘at-risk’ population [21]; this partly contributes to the widened 95% CI at 1 year.

Oliguria in ICU survivors was associated with a statistically significant increase in long-term mortality, even after sequential adjustment for potential confounders [fully adjusted HR 1.47 (95% CI 1.02–2.12), *P* = .038; *E*-value 1.93]. Mediation analysis suggested that this effect was partially mediated by length of stay (20.7%) and APACHE II score (18.4%), supporting the interpretation that these variables lie downstream of oliguria in the causal pathway and consistent with our DAGs. In contrast, vasopressor use and mechanical ventilation were included as proxies for haemodynamic compromise and impaired gas exchange, both key unmeasured determinants of renal oxygen delivery and oliguria. Although we lacked direct measurements of cardiac output, oxygen saturation and arterial oxygen tension, these proxies helped account for underlying illness severity without adjusting for downstream mediators. While this strengthens causal inference, proxy variables may imperfectly reflect the physiological states they represent. The *E*-value of 1.93 indicates that an unmeasured confounder would need to be associated with both oliguria and mortality by a HR of at least 1.93 to fully explain the observed effect. By integrating physiological understanding with causal modelling, our approach enhances the interpretability of effect estimates, although some residual confounding likely remains, as outlined in our DAGs.

When PBW was used instead of ABW, mortality lost statistical significance, likely reflecting two factors. First, PBW is less sensitive at detecting oliguric patients, hence we chose ABW from the outset to increase the sensitivity of our analyses [15]. Second, the reduction in sample size [from 1064 (ABW) to 708 (PBW) ICU survivors] causes analyses with PBW to lose statistical power. In analyses of patients that did not receive acute KRT, a similar loss of power and significance in 1-year mortality was seen due to the reduced sample size (from 1064 ICU survivors to 895 ICU survivors). Looking at a factor known to impact mortality (such as age [26]), the 95% CIs in logistic regression analyses widen from 1.02–3.82 (ABW) to 1.56–18.85 (PBW), showing a loss of precision, similar to what was seen with our CPH modelling, where the 95% CI widened from 1.02–2.06 (ABW) to 0.83–2.10 (PBW) due the smaller sample size [27].

No study to date has looked at outcomes beyond 28 days for oliguric AKI, therefore these results must be interpreted in isolation. Increased mortality in the oliguric cohort may partly be due to more severe illness seen in the oliguric cohort (suggested by their higher rates and longer durations of organ support, poorer APACHE II scores on admission and longer duration of critical care admission).

Oliguric AKI did not impact the likelihood of longer-term MAKEs occurring in ICU survivors. We assessed MAKEs in patients that survived to 30 days after hospital discharge, as we were interested in longer-term outcomes; however, this approach has the potential to miss patients who had a MAKE before this time point was reached (e.g. a patient discharged home having started long-term KRT). This was likely to have occurred, as the observed risk of MAKEs was significantly increased in the first 0.5 months of our stratified CPH model but lost significance from 0.5 to 12 months.

## Supplementary Material

sfaf170_Supplemental_Files

## Data Availability

Anonymised patient data collected during this study will be made available in the publicly available Enlighten repository (https://researchdata.gla.ac.uk). These data will be made available for a period of 10 years following completion of associated work towards attaining a thesis due to be completed no later than July 2023.
