# Supplementary material for: Short- and long-term outcomes in oliguric and non-oliguric acute kidney injury in intensive care: a retrospective, post hoc, bicentric study
Source: Clin Kidney J. 2025 May 30;18(6):sfaf170. doi: 10.1093/ckj/sfaf170 (PMC12203070; doi:10.1093/ckj/sfaf170)
Supplement: sfaf170_Supplemental_Files [file sfaf170_supplemental_files.zip › 908 suppl mat.docx]

**Supplementary Materials**: Short and long-term outcomes in oliguric and non-oliguric acute kidney injury in intensive care – a retrospective, post-hoc, bicentric study

**Authors**:

Sarjit Singh^1^

Mark Andonovic^1^

Jamie P Traynor^2^

Martin F Shaw^1^

Malcolm AB Sim^1,3^

Patrick B Mark^2,4^

Kathryn A Puxty^1,5^

**Affiliations**:

^1^Academic Unit of Anaesthesia, Critical Care and Perioperative Medicine, University of Glasgow

^2^Glasgow Renal and Transplant Unit, Queen Elizabeth University Hospital

^3^Department of Intensive Care, Queen Elizabeth University Hospital

^4^School of Cardiovascular and Metabolic Health, University of Glasgow

^5^Department of Intensive Care Medicine, Glasgow Royal Infirmary

**Correspondence to**:

Dr Sarjit Singh

Academic Unit of Anaesthesia, Critical Care and Peri-operative Medicine

Room 2.73, Level 2, New Lister Building

Glasgow Royal Infirmary

Glasgow G31 2ER

0141 956 0504

[sarjit.singh@glasgow.ac.uk](mailto:sarjit.singh@glasgow.ac.uk)

X: @sarjit94

ORCID: 0000-0001-5425-1807

**Supplementary Figure C1: Schoenfeld Residuals Plot for MAKE**


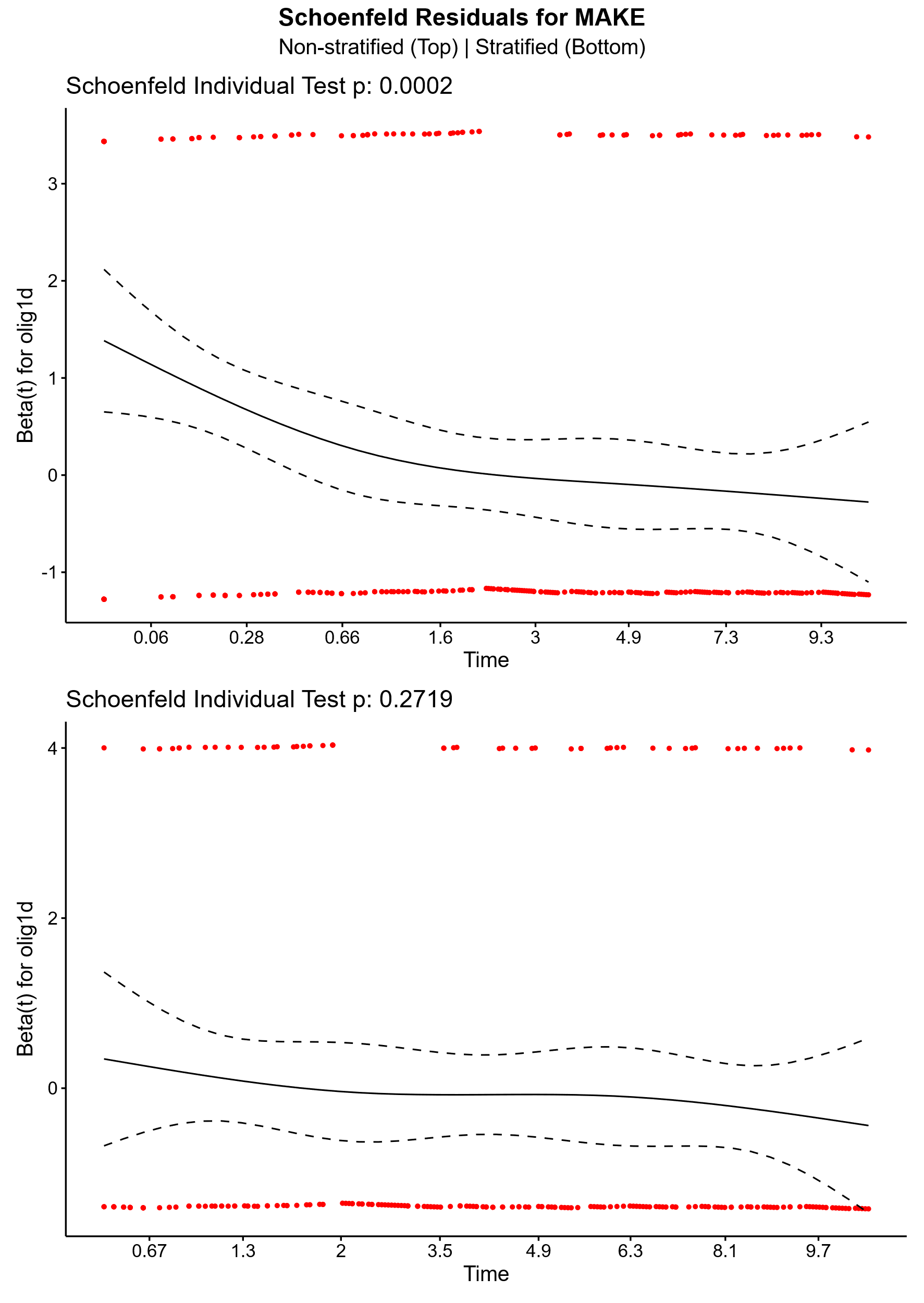


**Supplementary Figure R1**


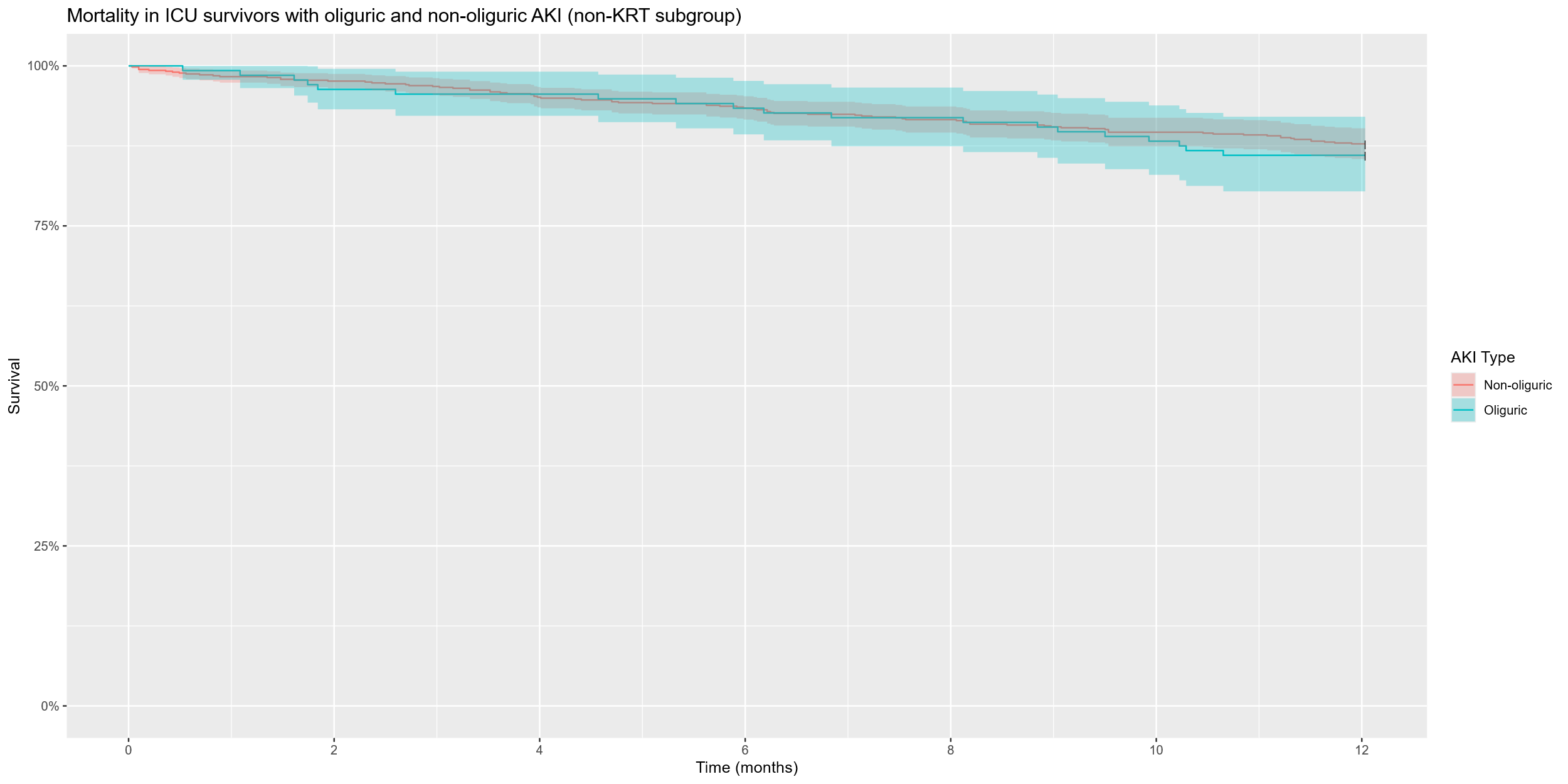


Mortality in ICU survivors with oliguric and non-oliguric AKI (non-KRT subgroup)

Alt text: A Kaplan-Meier plot showing overlap of the oliguric and non-oliguric cohorts over 1 year.

**Supplementary Figure R2**


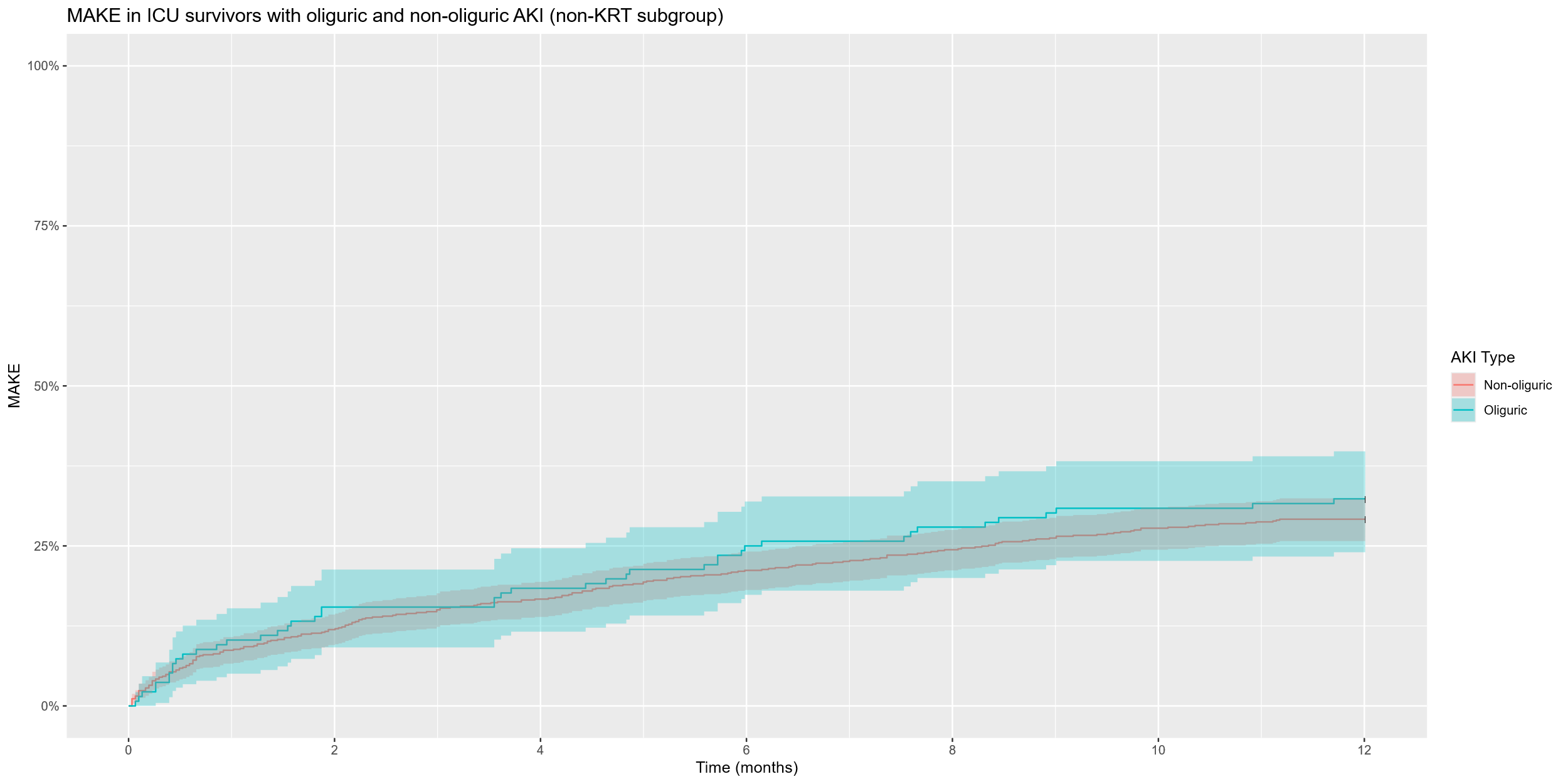


MAKE in ICU survivors with oliguric and non-oliguric AKI (non-KRT subgroup)

Alt text: A Kaplan-Meier plot showing overlap of the oliguric and non-oliguric cohorts over 1 year.

**Supplementary Figure W1**


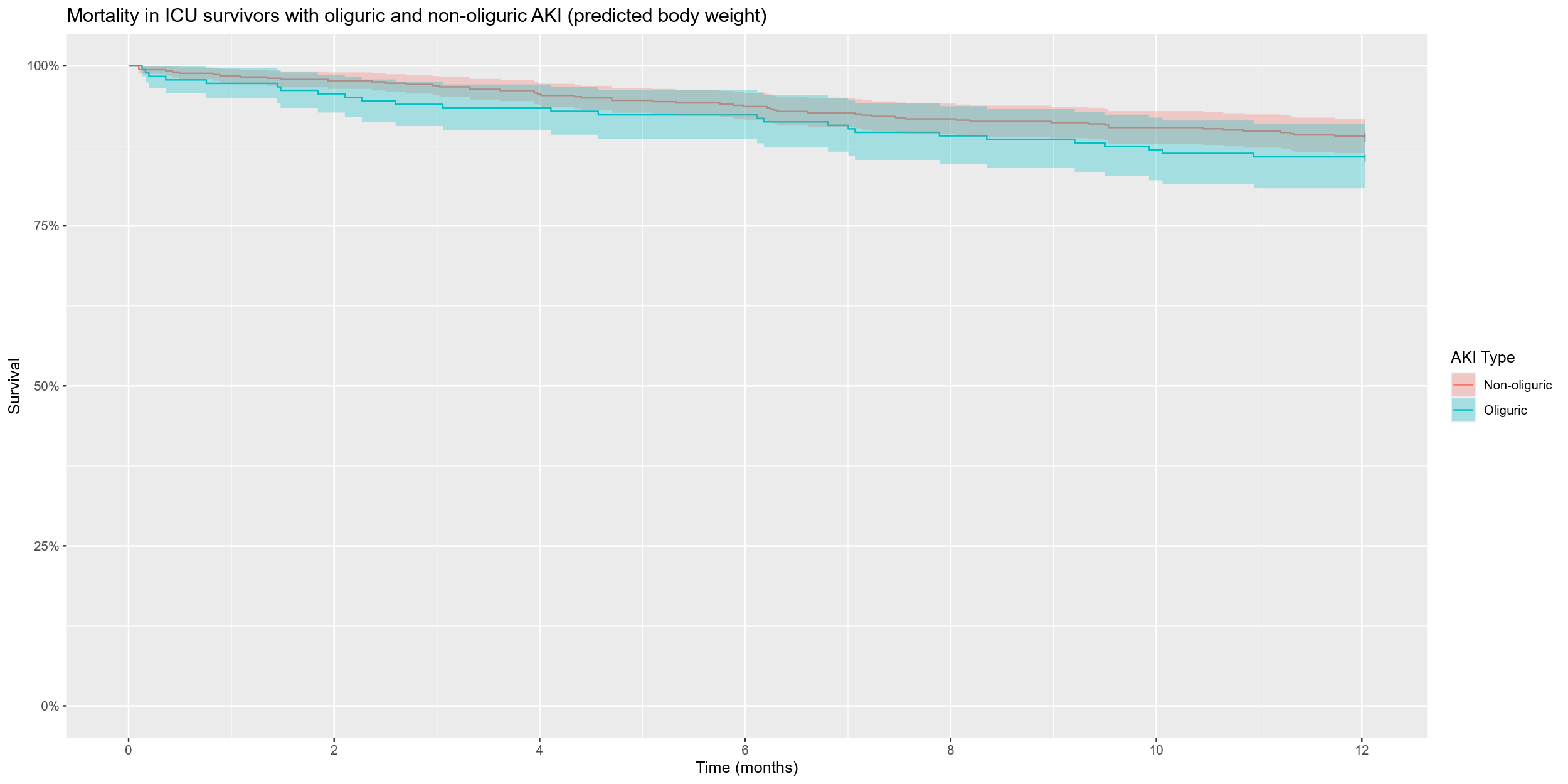


Mortality in ICU survivors with oliguric and non-oliguric AKI (predicted body weight)

Alt text: A Kaplan-Meier plot showing overlap of the oliguric and non-oliguric cohorts over 1 year. Survival is lower in the oliguric cohort at all points in the graph.

**Supplementary Figure W2**


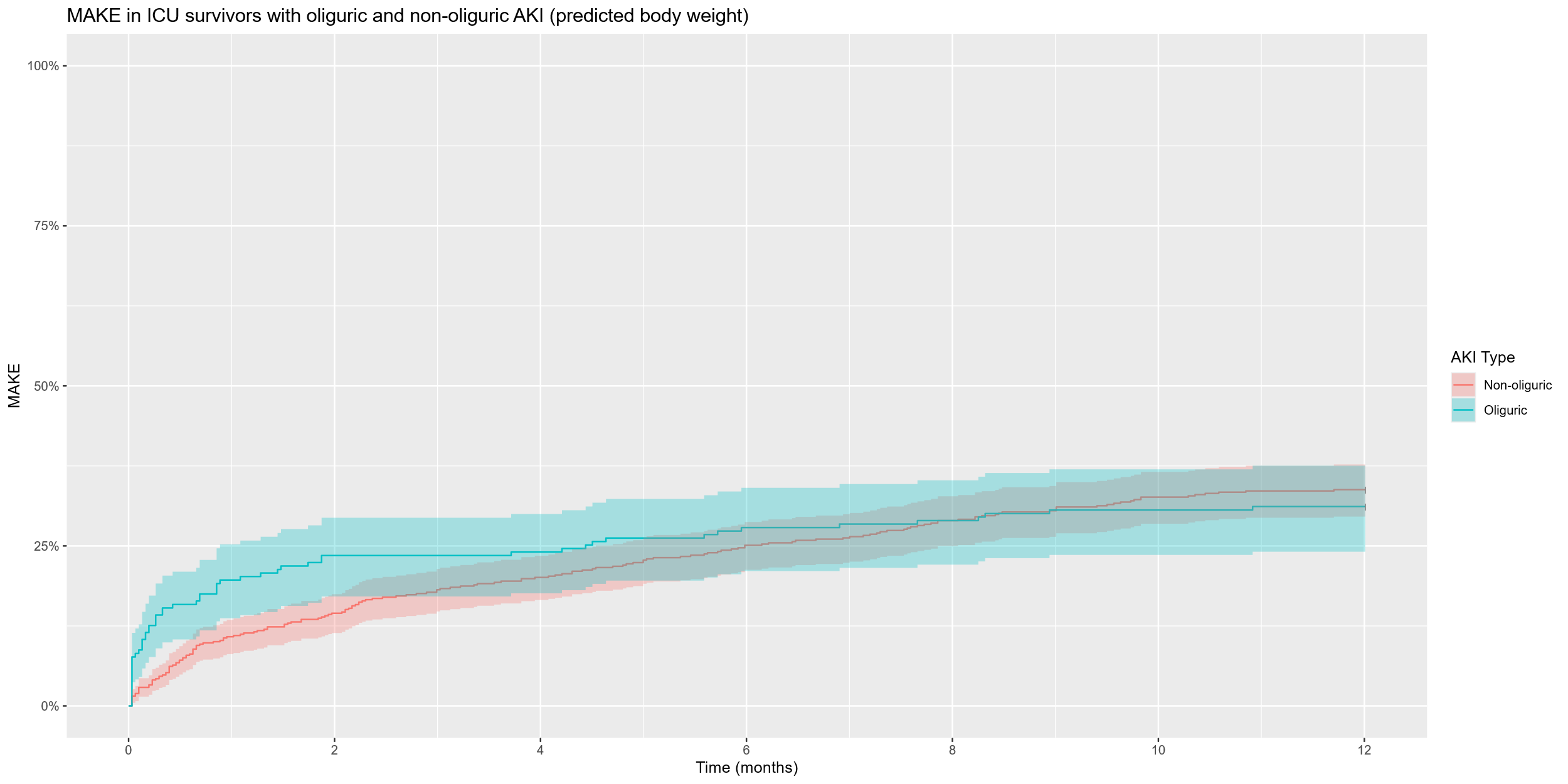


MAKE in ICU survivors with oliguric and non-oliguric AKI (predicted body weight)

Alt text: A Kaplan-Meier plot showing divergence and then crossing of the oliguric and non-oliguric cohorts over 1 year. Survival is lower in the oliguric cohort initially in the graph.

**Supplementary Table C1: stratified cox proportional hazards for MAKE**

|  | **Hazard Ratio** | **95% CI** | **p-value** |
| --- | --- | --- | --- |
| Oliguria  Non-oliguric  Oliguric | Ref  2.41 | 1.58 – 3.66 | <0.001 |
| Oliguria * period  Period 1  Period 2 | Ref  0.39 | 0.23 – 0.65 | < 0.001 |

**Supplementary Table C2: stratified cox proportional hazards for MAKE (isolated period 2)**

|  | **Hazard Ratio** | **95% CI** | **p-value** |
| --- | --- | --- | --- |
| Oliguria  Non-oliguric  Oliguric | Ref  0.94 | 0.70 – 1.26 | 0.7 |

**Table U1: Cohort demographics for patients excluding due to missing urine output data**

|  | **Overall**  **(n=2137)** | **Analysed**  **(n=1666)** | **Missing urine**  **output data**  **(n=471)** | **p-value** |
| --- | --- | --- | --- | --- |
| **Male** | 1,280 (59.9) | 1,001 (60.0) | 279 (61.0) | 0.847 |
| **Age** | 62 (49, 72) | 61 (49, 72) | 63 (48, 74) | 0.182 |
| **Baseline eGFR** | 82 (59, 99) | 83 (60, 99) | 80 (57, 99) | 0.279 |
| **Pre-existing co-morbidities** |  |  |  |  |
| **Cardiovascular Disease** | 962 (43.3) | 741 (45.0) | 221 (48.0) | 0.180 |
| **Respiratory Disease** | 436 (20.4) | 355 (21.0) | 81 (18.0) | 0.080 |
| **Liver Disease** | 234 (10.9) | 187 (11.0) | 47 (10.0) | 0.536 |
| **Diabetes Mellitus** | 401 (18.8) | 304 (18.0) | 97 (21.0) | 0.172 |
|  |  |  |  |  |
| **Admission from surgical specialty** | 1,098 (51.4) | 865 (52.0) | 233 (51.0) | 0.613 |
| **Admission with sepsis** | 632 (29.6) | 531 (32.0) | 101 (22.0) | <0.001 |
| **APACHE II score** | 22 (16, 28) | 22 (16, 29) | 19 (13, 26) | <0.001 |
|  |  |  |  |  |
| **ACP level 3 (days)** | 3 (1, 6) | 3 (1, 7) | 2 (1, 3) | <0.001 |
| **ACP level 2 (days)** | 1 (0, 2) | 1 (0, 3) | 1 (0, 2) | <0.001 |
| **Kidney support** |  |  |  |  |
| **Patients supported** | 492 (23.0) | 391 (23.0) | 101 (22.0) | 0.488 |
| **Days** | 8 (3, 16) | 8 (3, 17) | 2 (1, 3) | 0.130 |
| **Ventilatory support** |  |  |  |  |
| **Patients supported** | 1,570 (73.5) | 1,269 (76.0) | 301 (65.0) | <0.001 |
| **Days** | 12 (5, 23) | 12 (5, 23) | 2 (1, 4) | <0.001 |
| **Vasopressor support** |  |  |  |  |
| **Patients supported** | 1,443 (67.5) | 1,192 (72.0) | 251 (55.0) | <0.001 |
| **Days** | 6 (2, 15) | 6 (3, 15) | 2 (1, 3) | <0.001 |
|  |  |  |  |  |
| **MAKE** | 609 (28.5) | 455 (28.0) | 154 (34.0) | 0.013 |
| **Length of Kidney injury (days)** | 2 (1, 4) | 2 (1, 5) | 1 (1, 3) | <0.001 |
| **Length of ICU stay (days)** | 3 (2, 8) | 4 (2, 9) | 2 (1, 4) | <0.001 |
| **Length of hospital stay (days)** | 14 (5, 27) | 14 (5, 27) | 8 (2, 18) | <0.001 |
| **Death in ICU** | 627 (29.3) | 478 (29.0) | 149 (32.0) | 0.127 |
| **Death in hospital** | 751 (35.1) | 579 (35.0) | 172 (37.0) | 0.320 |

ACP: augmented care period; APACHE II: Acute Physiology and Chronic Health Evaluation II; eGFR: estimated glomerular filtration rate

**Table W1: Cohort Demographics**

|  | **Non-oliguric**  **(n=738)** | **Oliguric**  **(n=341)** | **p-value** |
| --- | --- | --- | --- |
| **Male** | 420 (56.9) | 213 (62.5) | 0.098 |
| **Age** | 62.00 [49.25, 72.00] | 63.00 [52.00, 71.00] | 0.902 |
| **Baseline eGFR** | 81.94 [61.01, 98.03] | 79.65 [51.37, 96.90] | 0.059 |
| **Pre-existing co-morbidities** |  |  |  |
| **Cardiovascular Disease** | 344 (46.6) | 163 (47.8) | 0.766 |
| **Respiratory Disease** | 161 (21.8) | 64 (18.8) | 0.287 |
| **Liver Disease** | 78 (10.6) | 40 (11.7) | 0.643 |
| **Diabetes Mellitus** | 127 (17.2) | 81 (23.8) | 0.014 |
|  |  |  |  |
| **Admission from surgical specialty** | 430 (58.3) | 195 (57.2) | 0.789 |
| **Admission with sepsis** | 243 (32.9) | 120 (35.2) | 0.508 |
| **APACHE II score** | 20.00 [15.00, 26.00] | 25.00 [20.00, 31.00] | <0.001 |
|  |  |  |  |
| **ACP level 3 (days)** | 2 [1, 6] | 5 [3, 13] | <0.001 |
| **ACP level 2 (days)** | 1 [0, 3] | 1 [0, 3] | 0.744 |
| **Kidney support** |  |  |  |
| **Patients supported** | 508 (68.8) | 272 (79.8) | <0.001 |
| **Days** | 2 [1, 2] | 5 [3, 10.25] | <0.001 |
| **Ventilatory support** |  |  |  |
| **Patients supported** | 502 (68.0) | 263 (77.1) | 0.003 |
| **Days** | 3 [2, 8] | 6 [3, 14] | <0.001 |
| **Vasopressor support** |  |  |  |
| **Patients supported** | 71 ( 9.6) | 200 (58.7) | <0.001 |
| **Days** | 2 [2, 4] | 4 [2, 8] | <0.001 |
|  |  |  |  |
| **MAKE** | 242 (33.1) | 77 (22.7) | 0.001 |
| **Length of Kidney injury (days)** | 2.00 [1.00, 3.00] | 5.00 [2.00, 16.00] | <0.001 |
| **Length of ICU stay (days)** | 3.00 [2.00, 7.00] | 7.00 [4.00, 14.00] | <0.001 |
| **Length of hospital stay (days)** | 14.00 [6.00, 27.00] | 17.00 [7.00, 31.50] | 0.004 |
| **Death in ICU** | 161 (21.8) | 119 (34.9) | <0.001 |
| **Death in hospital** | 205 (27.9) | 147 (43.4) | <0.001 |

ACP: augmented care period; APACHE II: Acute Physiology and Chronic Health Evaluation II; eGFR: estimated glomerular filtration rate

**Table W2: Logistic regression for development of oliguria**

|  | **Univariable** | | **Multivariable** | |
| --- | --- | --- | --- | --- |
|  | **OR (95% CI)** | **p-value** | **OR (95% CI)** | **p-value** |
| **Age**  <30 years  30 – 60 years  >60 years | Ref  1.32 (0.85 – 2.10)  1.32 (0.85 – 2.10) | 0.232  0.229 |  |  |
| **Sex**  Female  Male | Ref  1.26 (0.97 – 1.64) | 0.085 | Ref  1.27 (0.97 – 1.66) | 0.079 |
| **Baseline eGFR**  >60 ml min⁻¹ 1.73 m⁻²  30 – 60 ml min⁻¹ 1.73 m⁻²  <30 ml min⁻¹ 1.73 m⁻² | Ref  1.15 (0.82 – 1.60)  2.04 (1.28 – 3.22) | 0.399  0.002 | Ref  1.11 (0.79 – 1.55)  1.91 (1.19 – 3.04) | 0.548  0.007 |
| **Admitting specialty**  Medical  Surgical | Ref  0.95 (0.74 – 1.24) | 0.738 |  |  |
| **Admission diagnosis**  Non-sepsis  Sepsis | Ref  1.11 (0.84 – 1.45) | 0.464 |  |  |
| **Cardiovascular comorbidities**  Nil  Pre-existing diagnosis | Ref  1.05 (0.81 – 1.36) | 0.716 |  |  |
| **Pre-existing diabetes**  Nil  Pre-existing diagnosis | Ref  1.50 (1.09 – 2.05) | 0.012 | Ref  1.40 (1.02 – 1.93) | 0.039 |
| **Respiratory comorbidities**  Nil  Pre-existing diagnosis | Ref  0.83 (0.60 – 1.14) | 0.252 |  |  |
| **Pre-existing liver disease**  Nil  Pre-existing diagnosis | Ref  1.12 (0.74 – 1.68) | 0.570 |  |  |
| **Pre-existing cancer**  Nil  Pre-existing diagnosis | Ref  1.09 (0.68 – 1.72) | 0.717 |  |  |

eGFR: estimated glomerular filtration rate

**Table W3: Odds ratios for mortality**

|  | **Non-oliguric**  **(n=738)** | **Oliguric**  **(n=341)** | **p-value** | **OR [95% CI]** |
| --- | --- | --- | --- | --- |
| **Death in ICU** | 161 | 119 | <0.001 | 1.92 [1.45 – 2.55] |
| **Death in hospital** | 205 | 147 | <0.001 | 1.97 [1.51 – 2.58] |
| **Death within 30 days of hospital discharge** | 215 | 156 | <0.001 | 2.05 [1.57 – 2.68] |

**Table W4: Logistic regression for 1-year mortality**

|  | **Univariable** | | **Multivariable** | |
| --- | --- | --- | --- | --- |
|  | **OR (95% CI)** | **p-value** | **OR (95% CI)** | **p-value** |
| **AKI type**  Non-oliguric AKI  Oliguric AKI | Ref  1.33 (0.80 – 2.17) | 0.260 | Ref  1.27 (0.76 – 2.09) | 0.349 |
| **Age**  <30 years  30 – 60 years  >60 years | Ref  4.40 (1.55 – 18.45)  4.88 (1.72 – 20.53) | 0.015  0.010 | Ref  4.11 (1.45 – 17.29)  4.47 (1.56 – 18.85) | 0.021  0.015 |
| **Sex**  Female  Male | Ref  1.03 (0.65 – 1.66) | 0.894 |  |  |
| **Baseline eGFR**  >60 ml min⁻¹ 1.73 m⁻²  30 – 60 ml min⁻¹ 1.73 m⁻²  <30 ml min⁻¹ 1.73 m⁻² | Ref  1.07 (0.56 – 1.94)  1.09 (0.44 – 2.37) | 0.830  0.836 |  |  |
| **Admitting specialty**  Medical  Surgical | Ref  0.81 (0.51 – 1.30) | 0.379 |  |  |
| **Admission diagnosis**  Non-sepsis  Sepsis | Ref  1.17 (0.72 – 1.87) | 0.511 |  |  |
| **Cardiovascular comorbidities**  Nil  Pre-existing diagnosis | Ref  1.15 (0.72 – 1.82) | 0.558 |  |  |
| **Pre-existing diabetes**  Nil  Pre-existing diagnosis | Ref  1.79 (1.04 – 2.98) | 0.029 | Ref  1.73 (1.00 – 2.91) | 0.043 |
| **Respiratory comorbidities**  Nil  Pre-existing diagnosis | Ref  1.13 (0.63 – 1.93) | 0.678 |  |  |
| **Pre-existing liver disease**  Nil  Pre-existing diagnosis | Ref  0.95 (0.38 – 2.03) | 0.905 |  |  |
| **Pre-existing cancer**  Nil  Pre-existing diagnosis | Ref  1.76 (0.81 – 3.51) | 0.128 | Ref  1.77 (0.80 – 3.58) | 0.131 |

eGFR: estimated glomerular filtration rate

**Table W5: Logistic regression for 1-year MARE**

|  | **Univariable** | | **Multivariable** | |
| --- | --- | --- | --- | --- |
|  | **OR (95% CI)** | **p-value** | **OR (95% CI)** | **p-value** |
| **AKI type**  Non-oliguric AKI  Oliguric AKI | Ref  0.89 (0.61 – 1.27) | 0.515 | Ref  0.87 (0.59 – 1.28) | 0.494 |
| **Age**  <30  30 – 60  >60 | Ref  3.86 (2.05 – 7.93)  4.90 (2.60 – 10.09) | <0.001  <0.001 | Ref  3.20 (1.66 – 6.72)  3.83 (1.91 – 8.26) | 0.001  <0.001 |
| **Sex**  Female  Male | Ref  0.48 (0.35 – 0.66) | <0.001 | Ref  0.52 (0.37 – 0.72) | <0.001 |
| **Baseline eGFR**  >60  30 – 60  <30 | Ref  1.48 (0.97 – 2.23)  0.70 (0.36 – 1.29) | 0.066  0.272 | Ref  1.10 (0.70 – 1.72)  0.57 (0.28 – 1.10) | 0.662  0.107 |
| **Admitting specialty**  Medical  Surgical | Ref  0.86 (0.62 – 1.20) | 0.378 |  |  |
| **Admission diagnosis**  Non-sepsis  Sepsis | Ref  1.29 (0.93 – 1.79) | 0.132 | Ref  1.21 (0.85 – 1.70) | 0.290 |
| **Cardiovascular comorbidities**  Nil  Pre-existing diagnosis | Ref  1.48 (1.08 – 2.03) | 0.015 | Ref  1.17 (0.82 – 1.67) | 0.384 |
| **Pre-existing diabetes**  Nil  Pre-existing diagnosis | Ref  2.48 (1.69 – 3.65) | <0.001 | Ref  2.38 (1.58 – 3.59) | <0.001 |
| **Respiratory comorbidities**  Nil  Pre-existing diagnosis | Ref  1.11 (0.75 – 1.64) | 0.593 | Ref  0.88 (0.58 – 1.32) | 0.529 |
| **Pre-existing liver disease**  Nil  Pre-existing diagnosis | Ref  1.31 (0.76 – 2.23) | 0.326 | Ref  1.34 (0.75 – 2.36) | 0.317 |
| **Pre-existing cancer**  Nil  Pre-existing diagnosis | Ref  1.07 (0.59 – 1.89) | 0.812 |  |  |

eGFR: estimated glomerular filtration rate

**STROBE Statement**

|  | **Item No** | **Recommendation** | **Page No** |
| --- | --- | --- | --- |
| **Title and abstract** | 1 | (*a*) Indicate the study’s design with a commonly used term in the title or the abstract | 1 |
|  |  | (*b*) Provide in the abstract an informative and balanced summary of what was done and what was found | 2 |
| **Introduction** | | | |
| Background/rationale | 2 | Explain the scientific background and rationale for the investigation being reported | 4 |
| Objectives | 3 | State specific objectives, including any prespecified hypotheses | 4 |
| **Methods** | | | |
| Study design | 4 | Present key elements of study design early in the paper | 4-5 |
| Setting | 5 | Describe the setting, locations, and relevant dates, including periods of recruitment, exposure, follow-up, and data collection | 4-6 |
| Participants | 6 | (*a*) Give the eligibility criteria, and the sources and methods of selection of participants. Describe methods of follow-up | 4-5 |
|  |  | (*b*) For matched studies, give matching criteria and number of exposed and unexposed |  |
| Variables | 7 | Clearly define all outcomes, exposures, predictors, potential confounders, and effect modifiers. Give diagnostic criteria, if applicable | 4-6 |
| Data sources/ measurement | 8* | For each variable of interest, give sources of data and details of methods of assessment (measurement). Describe comparability of assessment methods if there is more than one group | 4-6 |
| Bias | 9 | Describe any efforts to address potential sources of bias | 5-6 |
| Study size | 10 | Explain how the study size was arrived at | ·· |
| Quantitative variables | 11 | Explain how quantitative variables were handled in the analyses. If applicable, describe which groupings were chosen and why | 5-6 |
| Statistical methods | 12 | (*a*) Describe all statistical methods, including those used to control for confounding | 6 |
|  |  | (*b*) Describe any methods used to examine subgroups and interactions | 5-6 |
|  |  | (*c*) Explain how missing data were addressed | 4 |
|  |  | (*d*) If applicable, explain how loss to follow-up was addressed | 4 |
|  |  | (*e*) Describe any sensitivity analyses | 5-6 |
| **Results** | | |  |
| Participants | 13* | (a) Report numbers of individuals at each stage of study—eg numbers potentially eligible, examined for eligibility, confirmed eligible, included in the study, completing follow-up, and analysed | 7 |
|  |  | (b) Give reasons for non-participation at each stage | 7 |
|  |  | (c) Consider use of a flow diagram | Fig1 |
| Descriptive data | 14* | (a) Give characteristics of study participants (eg demographic, clinical, social) and information on exposures and potential confounders | Tab1 |
|  |  | (b) Indicate number of participants with missing data for each variable of interest | Fig1 |
|  |  | (c) Summarise follow-up time (eg, average and total amount) | 6-7 |
| Outcome data | 15* | Report numbers of outcome events or summary measures over time | 6-7  Tab2 |

| Main results | 16 | (*a*) Give unadjusted estimates and, if applicable, confounder-adjusted estimates and their precision (eg, 95% confidence interval). Make clear which confounders were adjusted for and why they were included | 7  Tab3  Tab4 |
| --- | --- | --- | --- |
|  |  | (*b*) Report category boundaries when continuous variables were categorized | Tab3  Tab4 |
|  |  | (*c*) If relevant, consider translating estimates of relative risk into absolute risk for a meaningful time period | ·· |
| Other analyses | 17 | Report other analyses done—eg analyses of subgroups and interactions, and sensitivity analyses | 6-7  Tab5  Supl |
| **Discussion** | | | |
| Key results | 18 | Summarise key results with reference to study objectives | 8-9 |
| Limitations | 19 | Discuss limitations of the study, taking into account sources of potential bias or imprecision. Discuss both direction and magnitude of any potential bias | 10-11 |
| Interpretation | 20 | Give a cautious overall interpretation of results considering objectives, limitations, multiplicity of analyses, results from similar studies, and other relevant evidence | 8-10 |
| Generalisability | 21 | Discuss the generalisability (external validity) of the study results | 8-10 |
| **Other information** | | | |
| Funding | 22 | Give the source of funding and the role of the funders for the present study and, if applicable, for the original study on which the present article is based | 12 |

*Give information separately for exposed and unexposed groups.

**Note:** An Explanation and Elaboration article discusses each checklist item and gives methodological background and published examples of transparent reporting. The STROBE checklist is best used in conjunction with this article (freely available on the Web sites of PLoS Medicine at http://www.plosmedicine.org/, Annals of Internal Medicine at http://www.annals.org/, and Epidemiology at http://www.epidem.com/). Information on the STROBE Initiative is available at http://www.strobe-statement.org.

**Directed Acyclic Graphs**

APACHE-2 score


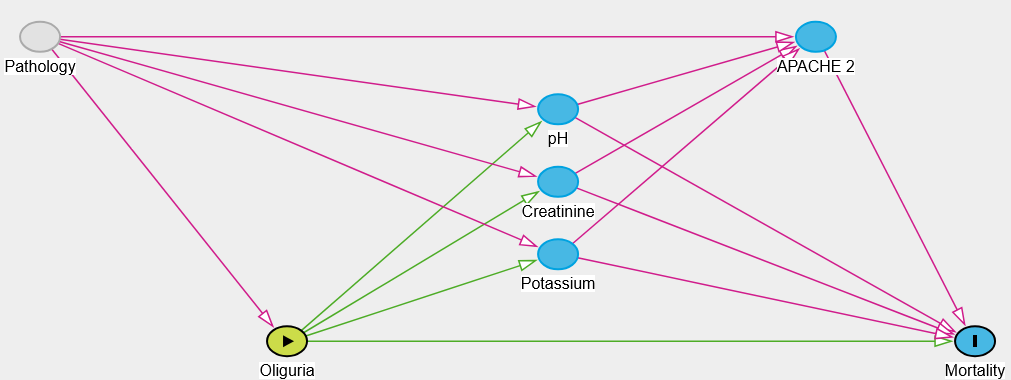


APACHE-2 score cannot be a confounder based on this DAG.

The score includes parameters such as pH, creatinine, and potassium, which are directly affected by oliguria; therefore, the arrows from oliguria must point toward these parameters. Since APACHE-2 incorporates these parameters, the arrows from them must, in turn, point toward the APACHE-2 score.

If APACHE-2 were a confounder in the relationship between oliguria and mortality, there would need to be an arrow from APACHE-2 to oliguria. This would create a cyclic relationship, which is not permitted in a DAG.

From a modelling perspective, this has important implications for adjustments. Because APACHE-2 lies downstream of oliguria and includes variables affected by it, adjusting for APACHE-2 would block part of the causal pathway from oliguria to mortality; this could lead to underestimation of the total effect. Moreover, if APACHE-2 also reflects downstream consequences of other processes, adjusting for it might introduce collider bias.

From this DAG, APACHE-2 score would be a mediator of the effect of oliguria on mortality; ergo, we have undertaken a formal mediation analysis.

Length of Stay


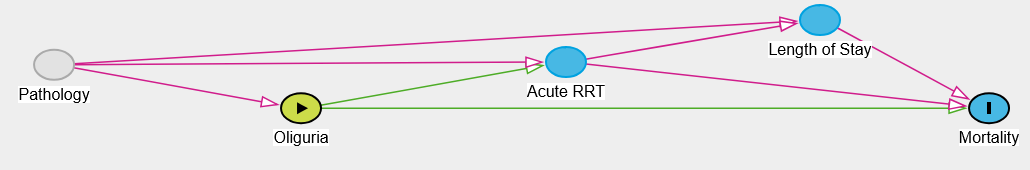


Length of stay cannot be a confounder based on this DAG.

It is reasonable to assume that oliguria influences the likelihood of receiving acute renal replacement therapy (RRT), which justifies an arrow from oliguria to acute RRT. Similarly, patients requiring acute RRT are likely to have longer hospital stays, supporting an arrow from acute RRT to length of stay.

For length of stay to function as a confounder in the relationship between oliguria and mortality, there would need to be a causal pathway from length of stay back to oliguria. Such a relationship would introduce a cycle, which violates the foundational principles of a DAG. Additionally, length of stay does not have the capacity to influence the underlying pathology that gives rise to oliguria. These considerations collectively reinforce that length of stay cannot act as a confounding variable in this context.

From this DAG, length of stay would be a mediator of the effect of oliguria on mortality; ergo, we have undertaken a formal mediation analysis.

Parameters affecting renal DO2

Renal oxygen delivery (DO₂) is a central physiological determinant of kidney function and, by extension, the risk of oliguria and mortality. Delivery of oxygen to the kidney is governed by the following relationship:

$$DO2=CO \times CaO2$$

$$CaO2=(1.34\times Hb \times SaO2)+(0.003\times PaO2)$$

$$DO2\propto CO \times[\left( Hb\times SaO2 \right)+PaO2]$$

Where:

- DO2 = oxygen delivery
- CO = cardiac output
- CaO2 = arterial oxygen content
- Hb = haemoglobin concentration
- SaO2 = arterial oxygen saturation
- PaO2 = arterial oxygen partial pressure
- 1.34 = Hüfner's constant
- 0.003 = solubility coefficient of O2 in plasma

From this relationship, both haemodynamic status (via CO) and gas exchange efficiency (via SaO2 and PaO2) are critical in determining renal oxygen delivery, and thereby influence the development of oliguria and the risk of mortality.


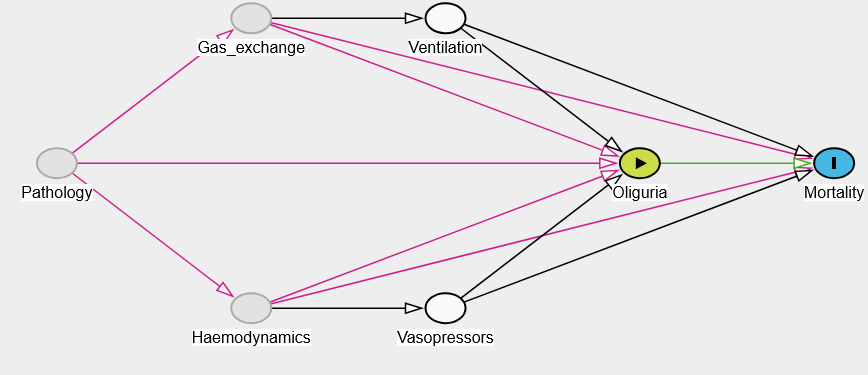


In our DAG, we illustrate how compromised haemodynamic (low CO) and impaired gas exchange (low SaO2 and PaO2) lead to reduced DO2. Clinically, vasopressors are used to support haemodynamic function and mechanical ventilation used to support oxygenation. As such, the use of vasopressors and mechanical ventilation can serve as proxies for these underlying physiological states and illness severity, which were not directly measured in our study.

In our fully adjusted model, we adjusted for vasopressor use and mechanical ventilation as surrogate markers of haemodynamic and gas exchange status, respectively. This adjustment strategy helps account for confounding by severity of illness, without controlling directly for unmeasured mediators on the causal pathway from underlying pathology to oliguria or mortality.

Importantly, vasopressor and ventilator use reflect the physiological condition (impaired perfusion or oxygenation) prior to or at the time of oliguria onset. These interventions are not consequences of oliguria but rather indicators of pre-existing illness severity that predisposes to it. Therefore, they function as proxies for pre-exposure confounding factors, rather than as post-exposure mediators. This approach supports causal inference by addressing confounding while avoiding inappropriate adjustment for downstream consequences of the exposure.

Fully adjusted DAG


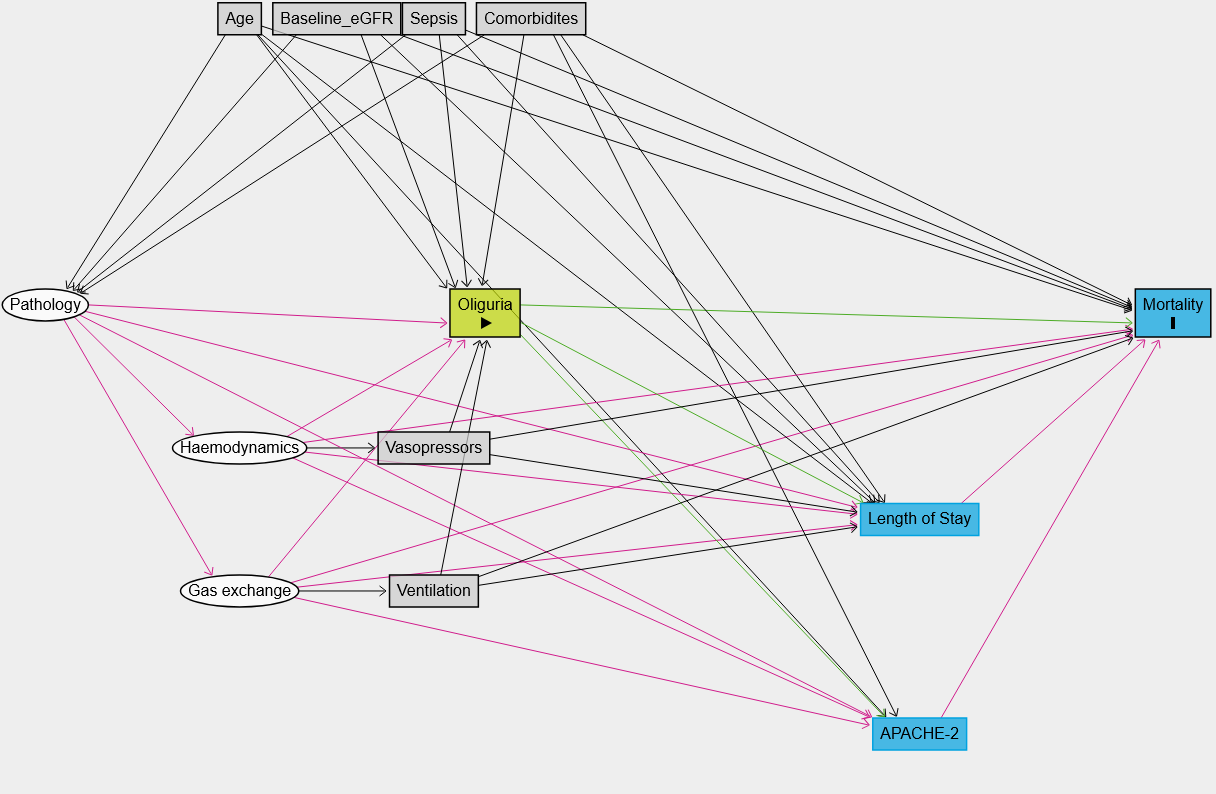


The fully adjusted DAG presented here integrates latent and observed variables to reflect a plausible causal structure linking oliguria to mortality, incorporating both physiological mechanisms and clinical interventions.

In this DAG, oliguria is the primary exposure, with mortality as the outcome. The causal pathway from oliguria to mortality is mediated by several downstream variables, most notably the APACHE-2 score and length of stay, both of which are conceptualized as mediators rather than confounders. Adjusting for these variables would therefore block part of the causal effect of interest and is avoided in our primary analysis.

Additionally, pathology is included as a latent variable that accounts for underlying disease processes (e.g., infection, inflammation, organ dysfunction) that may influence multiple downstream variables including haemodynamic, gas exchange, and oliguria.

This DAG structure allows us to adjust for confounding without inadvertently conditioning on colliders or mediators. It supports our approach to causal inference by explicitly mapping the hypothesized biological and clinical pathways and clarifying which variables are appropriate for adjustment. Our model reflects a physiologically informed view of acute kidney injury risk, framed by established determinants of renal oxygen delivery and systemic illness severity.

Nevertheless, some degree of residual bias is likely to persist. Not all relevant physiological or pathological processes are directly measured or fully captured by the variables available. Our use of vasopressors and ventilation as proxies helps mitigate this but cannot entirely account for the unmeasured complexity of illness severity. Similarly, misclassification or measurement error in recorded variables, as well as unmeasured confounders may introduce bias. While the DAG provides a framework for minimizing these sources of bias, it does not eliminate them entirely, and this should be acknowledged in the interpretation of effect estimates.
